# Association of dysbindin expression with individualized postoperative prognosis and chemotherapy benefit among patients with gastric adenocarcinoma

**DOI:** 10.7150/jca.60576

**Published:** 2021-09-21

**Authors:** Hao Qian, Xiaohui Lv, Qiying Song, Rujuan Su, Tianyu Xie, Di Wu, Rongyan Chang, Lubin Chen, Yanling Yang, Yong Chen, Xinxin Wang, Yi Ru, Lei Shang, Xin Guo

**Affiliations:** 1 Department of Health Statistics, School of Public Health, Fourth Military Medical University.; 2 Department of Endoscopic Surgery, Xijing Hospital, Fourth Military Medical University.; 3 Department of Gynecology and Obstetrics, Xijing Hospital, Fourth Military Medical University.; 4 Department of General Surgery, Chinese PLA General Hospital.; 5 Department of Oncology, Second Affiliated Hospital of Xi'an Jiaotong University.; 6 Department of Hepatobiliary Surgery, Xijing Hospital, Fourth Military Medical University.; 7 Department of Biochemistry and Molecular Biology, Fourth Military Medical University.

**Keywords:** dysbindin, overall survival, TNM stage, chemotherapy benefit

## Abstract

**Background:** The current model for predicting prognosis and chemotherapy response of patients with gastric adenocarcinoma is the TNM staging system, which may lack adequate accuracy and evaluations of molecular features at the individual level. We aimed to develop a prediction model to assess the individualized prognosis and responsiveness to fluorouracil-based adjuvant chemotherapy.

**Method:** This retrospective study concluded 2 independent cohorts of patients with GAC. The expression of dysbindin was quantified and evaluated the association with the overall survival for GAC patients. A prediction model for postoperative overall survival was generated and internally and externally validated. The interaction between dysbindin expression and PACT was detected in advanced GAC patients.

**Results:** Of the 637 patients enrolled in the study, 425 were men (66.7%) with a mean (SD) age of 59.79 (9.81) years. High levels of dysbindin expression predicted a poor prognosis in patients with GAC. Multivariate analysis demonstrated dysbindin expression was an independent prognostic predictor of overall survival in the test, validation and combined cohorts. A prognostic predictive model incorporating age, dysbindin expression, pathological differentiation, Lauren's classification and the TNM staging system was established. This model had better predictive accuracy for overall survival than the traditional TNM staging system and was internally and externally validated. More importantly, advanced GAC patients with low dysbindin expression were likely to benefit from fluorouracil-based PACT.

**Conclusion:** The risk stratification model incorporating dysbindin expression and TNM staging system showed better predictive accuracy. Advanced GAC patients with low dysbindin expression revealed better response of fluorouracil-based adjuvant chemotherapy.

## Introduction

Although the incidence and mortality rates of gastric adenocarcinoma (GAC) have declined in recent decades, in East Asian countries such as China, GAC remains the second most commonly diagnosed malignancy and the second leading cause of cancer-related death 1, 2. Currently, radical gastrectomy is generally advised as the only curative treatment for GAC 3. Unfortunately, the high rates of postoperative recurrence and metastasis results in poor overall survival (OS), making it critical to consider adjuvant treatments 4, 5. However, recent studies have indicated that GAC patients who received PACT based on fluorouracil regimens have only slightly improved OS rates, making the value of adjuvant chemotherapy controversial 6-9. Moreover, similar regimens of adjuvant chemotherapy in GAC patients with the same TNM stage result in a wide variety of clinical outcomes 10, 11. The current strategy for assessing risk stratification and prognosis is the TNM staging system, which may lack sufficient accuracy, especially in stage II and III GAC patients 12, 13. Consequently, an accurate risk stratification for GAC patients is warranted to improve the potential individual benefits obtained from postoperative chemotherapy.

Dysbindin, encoded by the dystrobrevin binding protein 1 gene, is a protein that functions as a component of the BLOC-1 complex, which is required for the normal biogenesis of lysosome-related organelles 14. In cancers, dysbindin is a novel oncoprotein that regulates the phosphorylation of the PI3K-Akt and ERK signaling pathways, which can lead to tumorigenesis and chemotherapy resistance 15, 16. In pancreatic ductal adenocarcinoma and ovarian cancer, dysbindin serves as an independent factor for prognosis and promoted invasion and metastasis 16, 17. However, the association of dysbindin expression and GAC remains unknown, and the underlying mechanism by which dysbindin acts in GAC requires further investigation.

In this study, we describe a novel model based on the TNM staging system and dysbindin expression that can be used to assess the individual risk and OS probability of patients with GAC. Furthermore, we aimed to explore whether the dysbindin expression status could be used to identify patients with advanced GAC who might sensitive to fluorouracil-based PACT.

## Materials and Methods

### Institutional Review Board Statement

The Medical Ethics Committee at each involved Chinese institutional medical center (Xijing Hospital, Fourth Military Medical University and the Chinese People's Liberation Army General Hospital) approved this study. All patients signed the informed consent form before their resected tissues were used.

### Patients and specimens

In this study, a test cohort of 375 consecutive GAC patients (male: female = 256: 119) was recruited from the Chinese People's Liberation Army General Hospital from October 2012 to May 2015 for model establishment and internal validation. An additional cohort of 262 consecutive patients (male: female =169:93) with the same diagnosis at Xijing Hospital from January 2013 to February 2015 was enrolled for external validation. The inclusion criteria were as follows: 1. the diagnosis of histologically confirmed GAC; 2. treatment with standard radical gastrectomy; 3. patients with advanced stage GAC were administered postoperative fluorouracil-based adjuvant chemotherapy for at least 4 cycle 18. Follow-up data needed to be available. The exclusion criteria were as follows: 1. preoperative anticancer treatments including chemotherapy, radiotherapy, immunotherapy or other cytotoxic therapy, and postoperative anticancer treatments in addition to routine chemotherapy; 2. the existence of distant metastasis; and 3. postoperative death due to complications.

The detailed clinicopathological characteristics of each patient, including gender, age, tumor size, tumor location, pathological differentiation, Lauren's classification and the TNM staging system were retrospectively collected. Pathological differentiation was classified as well, moderate, poor and undifferentiated according to the World Health Organization's gastric cancer treatment guidelines 19. The clinical staging was determined according to the 7^th^ edition of the American Joint Committee on Cancer (AJCC) and the International Union Against Cancer tumor-node-metastasis (TNM) staging system 20. OS refers to the time from the operation to the last follow-up or death. The median (range) follow-up time was 30.454 (2.8-87.2) months for the test cohort and 28.352 (3.9-88.5) months for the validation cohort. Data were analyzed between January 2020 and June 2020.

### Immunohistochemistry and evaluation of immunostaining

GAC and adjacent noncancerous tissues were obtained immediately after gastrectomy. Adjacent noncancerous tissues were defined as specimens adjacent to the margins of GAC (within 5cm). Before tissue microarray construction and immunohistochemistry, all specimens were sliced into 4 μm sections and stained by hematoxylin-eosin for accurate histological confirmation and selection of appropriate representative regions for each tissue. Immunohistochemical staining results were evaluated by two independent gastrointestinal pathologists who were blind to the study, and their results were averaged. Dysbindin expression was assessed with immunohistochemically staining according to the method described previously 17, 21. The immunohistochemical staining scores were determined as described previously by the following formula: intensity score × proportion score 22-24. Briefly, the indicated staining scores were applied for the corresponding intensities (0, no staining; 1, light yellow; 2, yellow brown; 3, strong brown color), and the indicated scores were applied for the corresponding proportions of positive tumor cells (0, 0% positive tumor cells; 1, 0%-10% positive tumor cells; 2, 10%-50% positive tumor cells; 3, 50%-100% positive tumor cells). The final immunoreactivity score (IS) for grouping was the product of the staining area score and the staining intensity. For the statistical analysis, the scores were grouped in two categories: scores of 0-3 were considered as low expression and 4-9 as high expression.

### Prediction model development and validation

Univariate and multivariate regression analyses were used to estimate the hazard ratio (HR) with 95% confidence intervals (CI) and determine the independent prognostic factors. The predictive model was constructed based on the known clinical prognostic factors and availability in the test cohort based on independent risk factors 25. After the test of Cox proportional hazards assumption, this model was implemented into the nomogram for predicting 3- and 5-year overall survival in GAC patients after surgery.

The predictive performance indicators of the nomogram include discrimination and calibration. Firstly, internal validation of the nomogram was performed with one thousand bootstrap resamples from the test cohort. The calibration curves were used to evaluate the goodness of fit between the predicted probabilities and observed outcomes. Secondly, the performance was further verified in the validation cohort and combined cohort to verify the applicability of the nomogram to other populations.

### Clinical use

In addition to the above verification methods, decision curve analysis (DCA) was further performed to compare the clinical usefulness and net benefit of the nomogram with those of the TNM staging system. It is generally considered that models with higher net benefit rates within a specific threshold range are more clinically useful 26.

### Statistical analysis

The continuous data are reported as the means ± SDs, and the differences between the cohorts were analyzed using independent-sample, unpaired, 2-tailed t tests or Mann-Whitney H tests, as appropriate. Categorical data are presented as proportions and percentages and were evaluated using the chi-square test or Fisher's exact test. Overall survival of patients' subgroups was compared by the Kaplan-Meier survival curve with the log-rank test. Univariate and multivariate regression analyses were applied to estimate the hazard ratio (HR) with 95% confidence intervals (CI) and identify the independent prognostic factor by Cox proportional hazards models. Interactions between the dysbindin expression and postoperative adjuvant chemotherapy treatment (PACT) were also detected by the Cox model. All statistical analyses were conducted with SPSS software (SPSS 26.0), RStudio software (RStudio, 1.2.5033) with R soft packages of “rms”, “time ROC”, and “stdca” and GraphPad Prism (GraphPad Prism, 8.1.244). Differences with two-sided P<0.05 were considered statistically significant. All authors had access to the study data and reviewed and approved the final manuscript.

## Results

### Participants

Our goal was to investigate whether dysbindin expression could serve as a prognostic and predictive indicator to identify patients at high risk and those likely to respond to chemotherapy. To this end, we conducted this study and the flow diagram is shown in Supplementary Fig. 1. The test cohort included of 375 consecutive patients, of whom 256 (68.3%) were men, with a median (interquartile range [IQR]) age of 57 (51-66) years. The validation cohort included of 262 consecutive patients, of whom 169 were men, with a median (interquartile range [IQR]) age of 58 (50-65) years. The clinicopathological characteristics of the patients in both cohorts were similar and given in Supplementary Table 1.

### Association of dysbindin expression with clinicopathological characteristics

**Figure 1:**
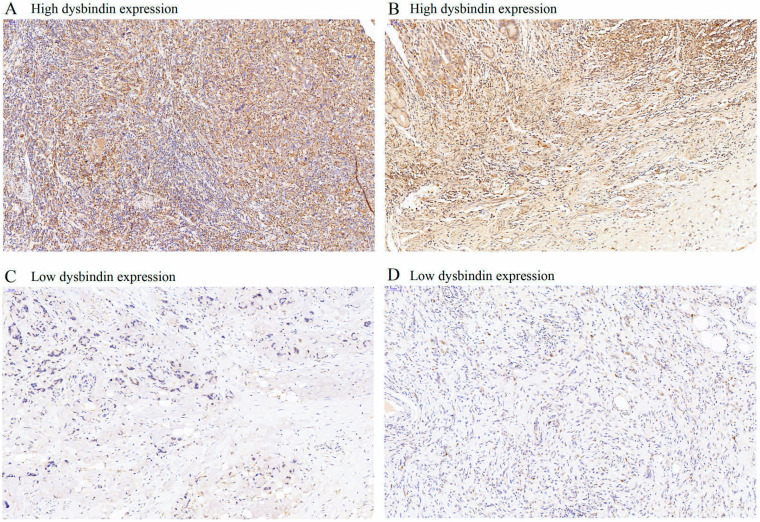
The expression levels of dysbindin in GAC tissues. **A and C,** Representative staining images of dysbindin high expression. **B and D,** Representative staining images of dysbindin low expression. All specimens were stained by immunohistochemistry assays with an original magnification of ×200.

Dysbindin expression was immunohistochemically detected in 637 patients with GAC. Dysbindin expression was high in GAC tissues but low or absent in adjacent noncancerous tissues (Fig. 1). GAC tissues were classified into two groups according to dysbindin expression levels. In the test cohort, 251 specimens (66.9%) had high expression levels of dysbindin, and 124 (33.1%) had low levels. In the validation cohort, 168 specimens (64.1%) had high expression levels of dysbindin, and 94 (35.9%) had low levels. In addition, no significant correlation was detected between dysbindin expression and the clinicopathological characteristics of patients with GAC, which are shown in Supplementary Table 2.

### Association of dysbindin expression and the prognosis of patients with gastric adenocarcinoma

In order to explore the prognostic value of dysbindin in patients with GAC, we performed Kaplan-Meier survival curves to compare the OS of patients with different levels of dysbindin expression. In the test cohort (HR, 2.516; 95% CI, 1.890-3.349; P<0.0001), validation cohort (HR, 3.749; 95% CI, 2.659-5.286; P<0.0001) and combined cohort (HR, 2.947; 95% CI, 2.366-3.671; P<0.0001), patients with high dysbindin expression had poorer OS than those with low dysbindin expression (Supplementary Fig. 2).

Then, we performed a stratified analysis of patients with GAC according to their clinicopathological characteristics. In test, validation and combined cohorts, patients with high dysbindin expression both had shorter OS time than patients with low dysbindin expression in AJCC stage I, II and III (Supplementary Fig. 3). Furthermore, dysbindin expression remained a powerful prognostic predictor after stratification by age, gender, tumor size, tumor location, Lauren's classification, pathological differentiation, T stage and N stage (Supplementary Fig. 4-6).

Finally, as shown by univariable analysis, patients with high expression levels of dysbindin were associated with significantly poorer OS (Table 1). Furthermore, multivariate Cox regression analysis revealed that dysbindin expression and the TNM staging system were both identified as the independent prognostic factors in GAC patients (Table 1 and Supplementary Table 3-4). Hence, dysbindin expression may serve as a reliable and independent and dangerous prognosticator of OS for patients after GAC surgery.

### Prognostic power for dysbindin expression and the TNM staging system

**Figure 2:**
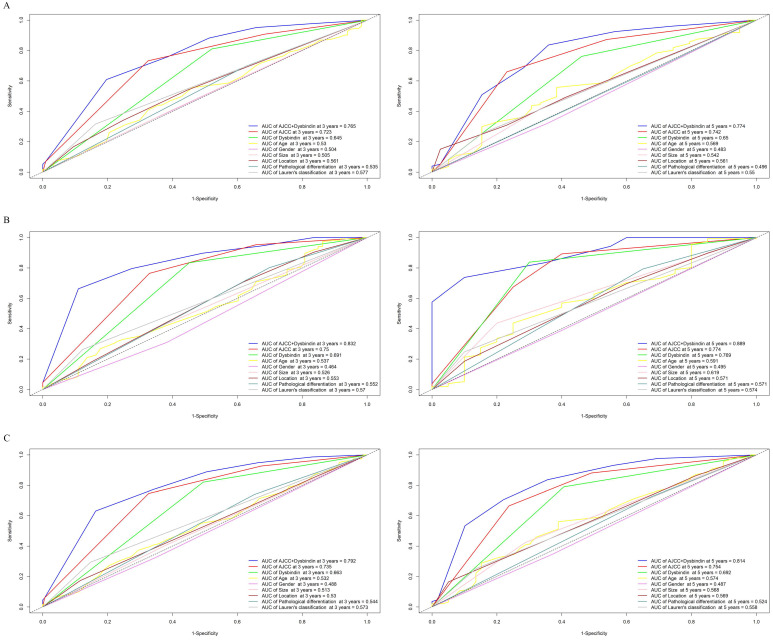
Time-dependent receiver operating characteristic (ROC) curves for assessing dysbindin expression, TNM stage and clinicopathological characteristics as predictors of 3- and 5-year OS in different cohorts. A, the 3- and 5-year ROC curves in test cohort. B, the 3- and 5-year ROC curves in validation cohort. C, the 3- and 5-year ROC curves in combined cohort. The AUC value of each predictor was shown in the figure.

After the expression of dysbindin and the TNM staging system were confirmed as two independent prognostic risk factors, we then tried to evaluate whether integrating the dysbindin expression and the current TNM staging system will improve the prognostic predictive ability. As shown in the Fig. 2 and Supplementary Table 5, the AUCs of dysbindin expression were lower than those of the TNM staging system. However, the integration of dysbindin expression and the TNM staging system showed better prognostic accuracy for 3- and 5- year OS (test cohort: 0.765, 0.774; validation cohort: 0.832, 0.889; combined cohort: 0.792, 0.814) than the TNM staging system, dysbindin expression or any other clinicopathological characteristics alone. These results suggest that dysbindin expression may complement the TNM staging system in the prognostic prediction after surgery.

### Development and assessment of an individualized prognostic prediction nomogram

**Figure 3:**
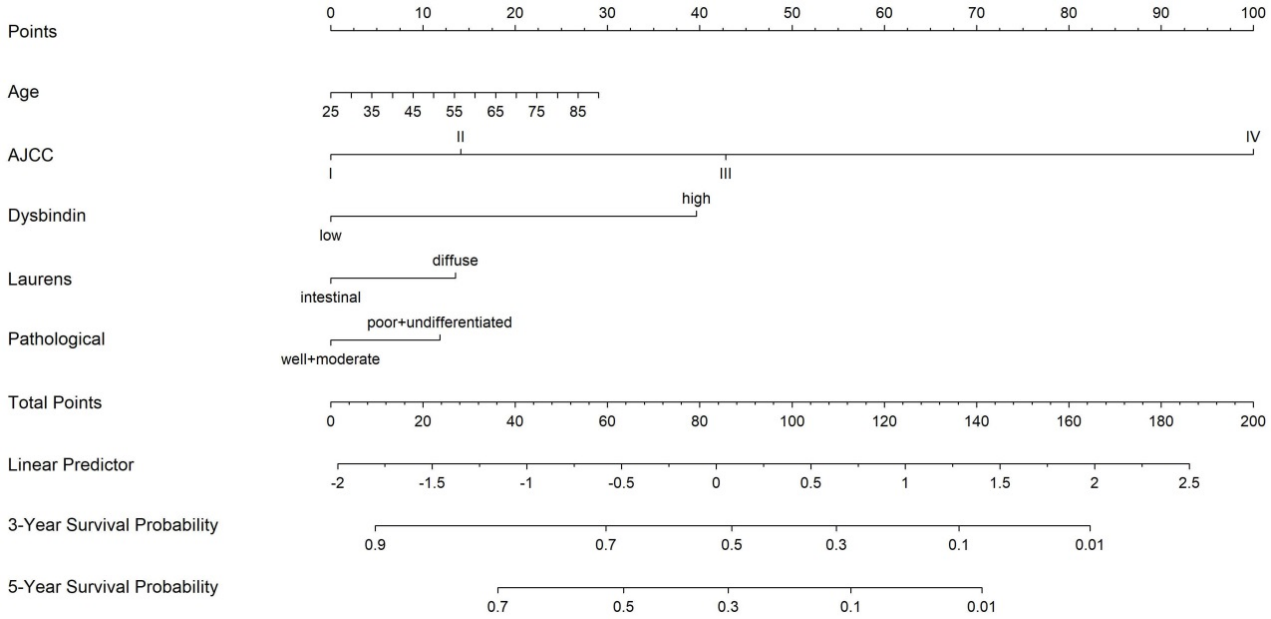
A nomogram to predict 3- and 5-year overall survival rates after D2 gastrectomy for GAC patients. The nomogram is used by summing the points identified on the point scale for each variable. The total points projected on the bottom scales indicate the probability of 3- and 5-year OS.

To establish a more effective prognostic model, we constructed a Cox model on the basis of the independent prognostic risk factors and several known clinical prognostic factors. Included covariates were age, TNM staging system, dysbindin expression, Lauren's classification, pathological differentiation. The test of Cox proportional hazards assumption of the model showed that each covariates test and global test were P > 0.05, which did not violate the assumption of proportional hazard (Supplementary Fig. 7 and Supplementary Table 6). Then, the Cox model was implemented into the nomogram to predict the overall survival at 3 and 5 years for GAC patients after surgery. Thus, a nomogram incorporating age, pathological differentiation, Lauren's classification, dysbindin expression and the TNM staging system was developed (Fig. 3).

The performance of the nomogram was verified by discrimination and calibration. This new prediction model showed adequate accuracy for the prediction of OS at 3 and 5 years in patients after GAC operation, supported by the calibration curves which revealed good consistency between predicted and observed outcomes (Supplementary Fig. 8) in the test cohort, validation cohort and combined cohort. The results of the comparation between the nomogram and the TNM staging system showed that the C-index of the nomogram (test cohort, 0.720; validation cohort, 0.718; combined cohort, 0.719) was significantly higher than those of the TNM staging system (test cohort, 0.646; validation cohort, 0.632; combined cohort, 0.641) (Supplementary Table 7). The time-dependent ROC of the nomogram also showed better prediction accuracy than that of the TNM staging system (Supplementary Fig. 9). Furthermore, the same results were obtained for each time period in the time-dependent AUC analysis in test and combined cohorts (Supplementary Fig. 10). These results suggest that the novel nomogram had a higher predictive ability than the traditional TNM staging system.

Finally, we also performed decision curve analysis (DCA) to assess the clinical value of this nomogram. As shown in Supplementary Fig. 11, the net benefit rate of the nomogram is better than that of the TNM stage at 3- and 5-year with a large threshold (Pt) range (0.2-0.7). In addition, the DCAs of the validation cohort and combined cohort also showed that the nomogram had a higher net benefit than that of the TNM staging system, regardless of whether it was evaluated at 3-year or 5-year.

### Correlation between dysbindin expression and postoperative adjuvant chemotherapy benefit

Although several classic clinical trials have indicated that patients with GAC derive a survival benefit from postoperative adjuvant chemotherapy, the chemotherapy response varies widely among patients with stage II or III GAC. Thus, we further investigated the correlation between dysbindin expression and the benefit derived from chemotherapy. An interaction test showed that patients with stage II GAC benefited more from adjuvant chemotherapy if they had low dysbindin expression levels than if they had high expression levels (HR, 0.3526; 95% CI, 0.1152-1.0790; P=0.0321 for interaction). However, patients with stage III GAC showed no significant differences between patients with high dysbindin expression and those with low dysbindin expression (HR, 0.5056; 95% CI, 0.2677-0.9547; P=0.5720 for interaction) in Table 2. The Kaplan-Meier survival curves for patients with stage II or stage III GAC, which comprehensively compared the patients with low and high dysbindin levels stratified according to treatment stratification, were shown in Supplementary Fig. 12. These results demonstrated that stage II patients with low level of dysbindin expression and all stage III patients could benefit more from fluorouracil-based postoperative adjuvant chemotherapy.

## Discussion

GAC is a highly heterogeneous disease with various difference in clinical outcomes. Thus, accurate prognostic evaluation is very important when determining the appropriate treatment. The traditional model for prognostic risk stratification and postoperative treatment determination in patients with GAC is based on the TNM staging system. However, this existing model has limitations due to the lack of sufficient accuracy even among patients with the same TNM stage. Recently, treatment based on molecular tumor features has offered an increasingly promising approach to therapy decision-making in patients with lung cancer and colorectal cancer 27-30. In our study, we identified dysbindin expression as the independent prognostic predictive factor in patients with GAC by Cox analysis. The ROC curves showed that the integration of dysbindin expression and TNM staging system had better predictive accuracy than the TNM staging system and other clinicopathological characteristics alone. Thus, a novel predictive model incorporating the TNM staging system and dysbindin expression was developed. This nomogram showed better performance on prognostic accuracy than those of TNM staging system.

To our knowledge, the most pressing concern of patients with GAC is the duration of postoperative survival. To this end, we constructed this nomogram to predict of 3-year and 5-year overall survival. This nomogram could provide surgeons and patients with not only a general prognosis at the time of diagnosis but also information that can be used to select appropriate treatment. In addition, dysbindin expression could be used to classify patients within each stage into high- and low-risk groups and allow surgeons to identify potential candidates for systemic treatment to improve outcomes. Therefore, this nomogram may be a valid and useful tool for clinicians in routine clinical practice. Patients with GAC may choose to undergo systemic treatments to improve outcomes on the basis of this nomogram and their dysbindin expression levels.

Several classic clinical trials have indicated GAC patients derive a survival benefit from postoperative adjuvant chemotherapy, which has led to it being widely recommended as standard therapy, especially for stage II and III patients 31, 32. However, there have been large variations in chemotherapy response and clinical outcomes 33. Hence, identifying patients who likely to be sensitive to chemotherapy will not only improve outcomes but also reduce excessive toxicities. Currently, fluorouracil is generally used as a mainstream chemotherapeutic drug. In this study, we assessed the association between dysbindin expression and the OS of patients with stage II and III GAC who received fluorouracil-based postoperative advanced chemotherapy. The results suggested that patients with low level of dysbindin expression were more likely to derive a survival benefit from PACT than those with high dysbindin expression levels. This strongly indicated that dysbindin expression could be a promising predictive factor for the response to chemotherapy. To our knowledge, no study has reported the relationship between the expression of dysbindin and chemotherapy response. This new molecular feature of GAC may help surgeons select and manage candidates for fluorouracil-based chemotherapy.

In recent years, increasing attention has been paid to precise molecular tumor features in terms of survival prediction and the potential response to therapy. LncRNA MNX1-AS1 has been reported to be upregulated in gastric cancer, and its upregulation indicates poor prognosis 34. Another lncRNA panel has been reported to predict the chemotherapy response of early-stage colorectal cancer 35. Wang et al. reported that lncRNA-ROR regulated multidrug resistance genes and predicted poor prognosis in patients with gastric cancer 36. To our knowledge, although these signatures showed promising results with regard to assessing the response to chemotherapy, these tests were mainly based on RT-PCR, which requires high technical proficiency and skilled operators. However, IHC has been widely applied in routine clinical tests, which provide not only quantitative analyses of target proteins but also their accurate cellular localization. Hence, we believe that the identification of dysbindin expression in GAC with IHC could be a stable and routine molecular profiling test that could be used to predict the response of GAC to chemotherapy.

In this study, we developed a novel nomogram including age, pathological differentiation, Lauren's classification, TNM stage and dysbindin expression. This nomogram showed a satisfactory predictive ability for OS, as reflected in the C-index value and AUC. More importantly, this nomogram retained its predictive value after internal bootstrap resampling and external validation in a cohort from another hospital, as recommended by statisticians 37. We believed that the selection bias could be minimized with internal bootstrap resampling. Furthermore, the transferability and generalizability of this nomogram were confirmed in an independent external cohort. Thus, the newly developed model showed promising predictive value for individualized risk stratification and OS.

Several advantages of this nomogram were also observed. First, the predictions made using this nomogram could inform the initial postoperative OS assessment. GAC patients with a poor prognosis according to this nomogram may need more proactive treatment, which may not be limited to chemotherapy. Second, this nomogram could identify patients who are likely to be sensitive to fluorouracil-based chemotherapy based on individualized molecular features. Since chemotherapy is recommended for patients with advanced GAC, we think this is very important to avoid ineffective chemotherapy and identify the candidates who are likely to be sensitive to fluorouracil. Third, using this nomogram, the follow-up plan for patients with poor predicted prognosis could be formulated with consideration of that prediction. Clinicians may be able to provide more accurate prospective evaluations to these patients. Fourth, the specific characteristics incorporated in this nomogram were simple and readily available, and even the pathological factors were routinely obtained in clinical practice. Fifth, the decision curve constructed based on this nomogram may facilitate the assessment by clinicians of the net clinical benefits of treatment.

Although this nomogram showed promising predictive value, this study has several limitations. Firstly, its retrospective nature limited the study to some extent, and the number of patients polled was still relatively small. Secondly, the OS of patients was affected by not only GAC but also comorbidities, which were not reflected in this nomogram. It is complicated to categorize and quantify those variables. Third, this nomogram did not account for the impact of race on OS. All the participants enrolled in this study were Asian, which may limit the widespread applicability of the nomogram because of the different treatment strategies and possible variations due to differences among ethnicities.

In summary, this study showed that the expression level of dysbindin is an independent prognostic predictive risk factor in patients with GAC. The newly developed nomogram incorporating the TNM staging system and dysbindin expression showed promising prognostic predictive capacity after internally and externally validation. More importantly, dysbindin expression could be used to identify a subset of patients with advanced GAC who are likely to be sensitive to fluorouracil-based postoperative advanced chemotherapy. These findings may shed new light on individualized precision therapy for GAC. However, the clinical value of this nomogram still needs further confirmation in a prospective study.

## Supplementary Material

Supplementary figures and tables.

## Figures and Tables

**Table 1:** Univariate and multivariate Cox regression analysis of patients with GAC in test cohort

Factors	No	Overall survival			
		Univariate analysis		Multivariate analysis	
		HR (95% CI)	*P* value	HR (95% CI)	*P* value
**Dysbindin expression**			<0.001		<0.001
Low	124	1 (Reference)		1 (Reference)	
High	251	2.547 (1.811-3.581)		2.544 (1.803-3.590)	
**Gender**			0.382		
Male	256	1 (Reference)			
Female	119	1.142 (0.848-1.538)			
**Age (years)**			0.007		0.125
≤60	229	1 (Reference)		1 (Reference)	
>60	146	1.017 (1.005-1.030)		1.010 (0.997-1.022)	
**Size (cm)**			0.591		
≤4	225	1 (Reference)			
>4	150	1.082 (0.811-1.443)			
**Tumor location**			0.558		
Cardia	75	1 (Reference)			
Body	61	1.074 (0.667-1.729)			
Antrum	189	0.971 (0.662-1.425)			
Whole	50	1.310 (0.802-2.138)			
**Pathological differentiation**			0.113		
Well + moderate	128	1 (Reference)			
Poor + undifferentiated	247	1.284 (0.942-1.751)			
**Lauren's classification**			0.126		
Intestinal type	272	1 (Reference)			
Diffuse type	103	1.282 (0.933-1.762)			
**TNM stage**			<0.001		<0.001
I	58	1 (Reference)		1 (Reference)	
II	91	1.550 (0.911-2.638)		1.440 (0.844-2.457)	
III	213	3.158 (1.971-5.059)		2.873 (1.787-4.619)	
IV	13	12.035 (5.962-24.294)		12.351 (5.973-25.543)	

**Table 2:** Treatment interaction with dysbindin expression for overall survival

Dysbindin expression	Chemotherapy	No chemotherapy	HR (95% CI)	P value for interaction
**AJCC stage II GAC**				
High expression group (n=93)	48	45	0.7052 (0.3999-1.2430)	0.0321
Low expression group (n=54)	30	24	0.3526 (0.1152-1.0790)	
**AJCC stage III GAC**				
High expression group (n=258)	141	117	0.6608 (0.4861-0.8984)	0.5720
Low expression group (n=121)	50	71	0.5056 (0.2677-0.9547)	

^a^ included stage II and III patients with GAC.

## Abbreviations

GAC: gastric adenocarcinoma
IHC: immunohistochemistry
TNM: tumor-node-metastasis
HR: hazard ratio
CI: confidence interval
PACT: postoperative adjuvant chemotherapy treatment
DCA: decision curve analysis
ROC: receiver operating characteristic
