# Supplementary material for: Association of dysbindin expression with individualized postoperative prognosis and chemotherapy benefit among patients with gastric adenocarcinoma
Source: J Cancer. 2021 Sep 21;12(22):6740–8. doi: 10.7150/jca.60576 (PMC8518004; doi:10.7150/jca.60576)
Supplement: Supplementary file 1 — Supplementary figures and tables. [file jcav12p6740s1.pdf]

Supplementary Figure 1

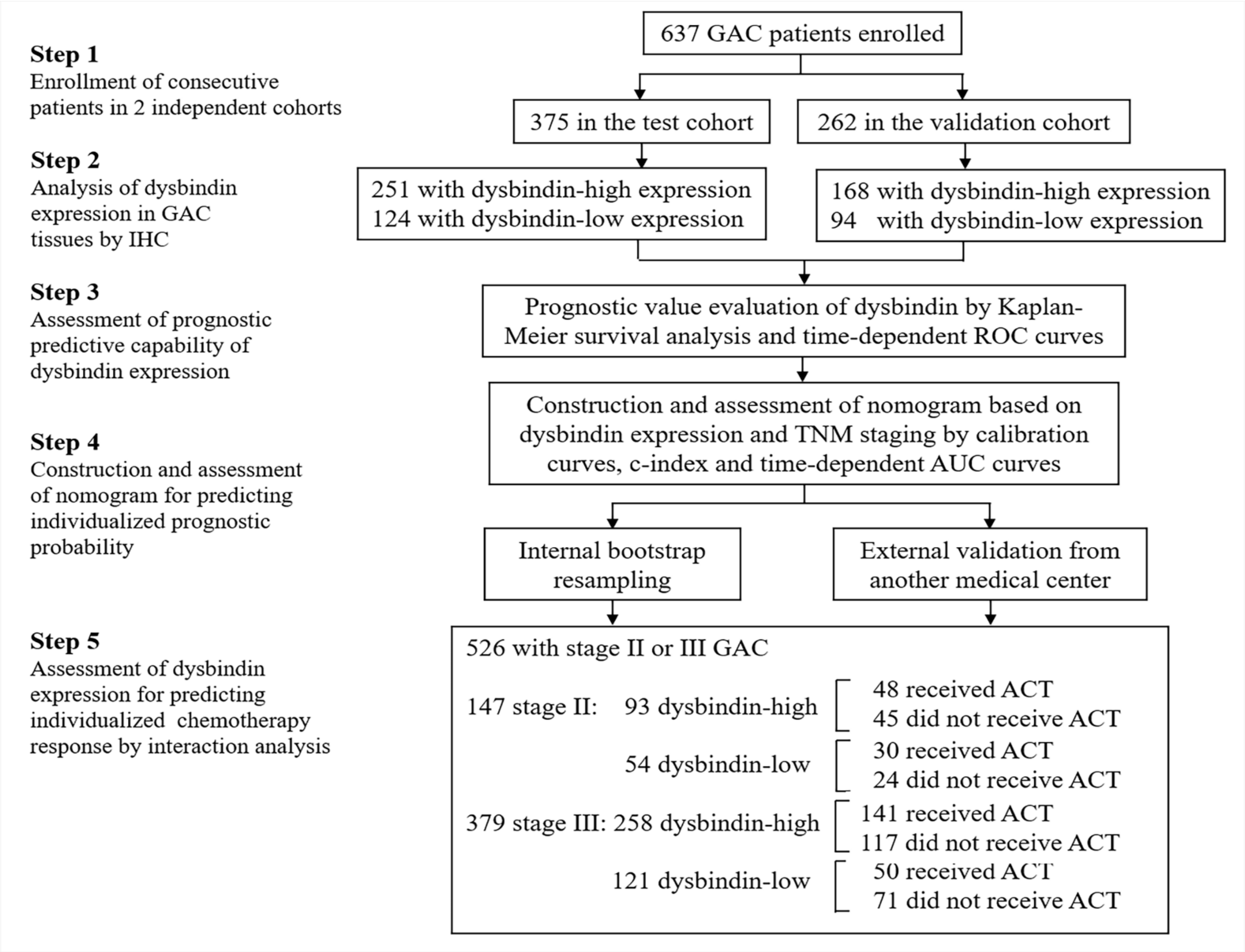

Supplementary Figure 2

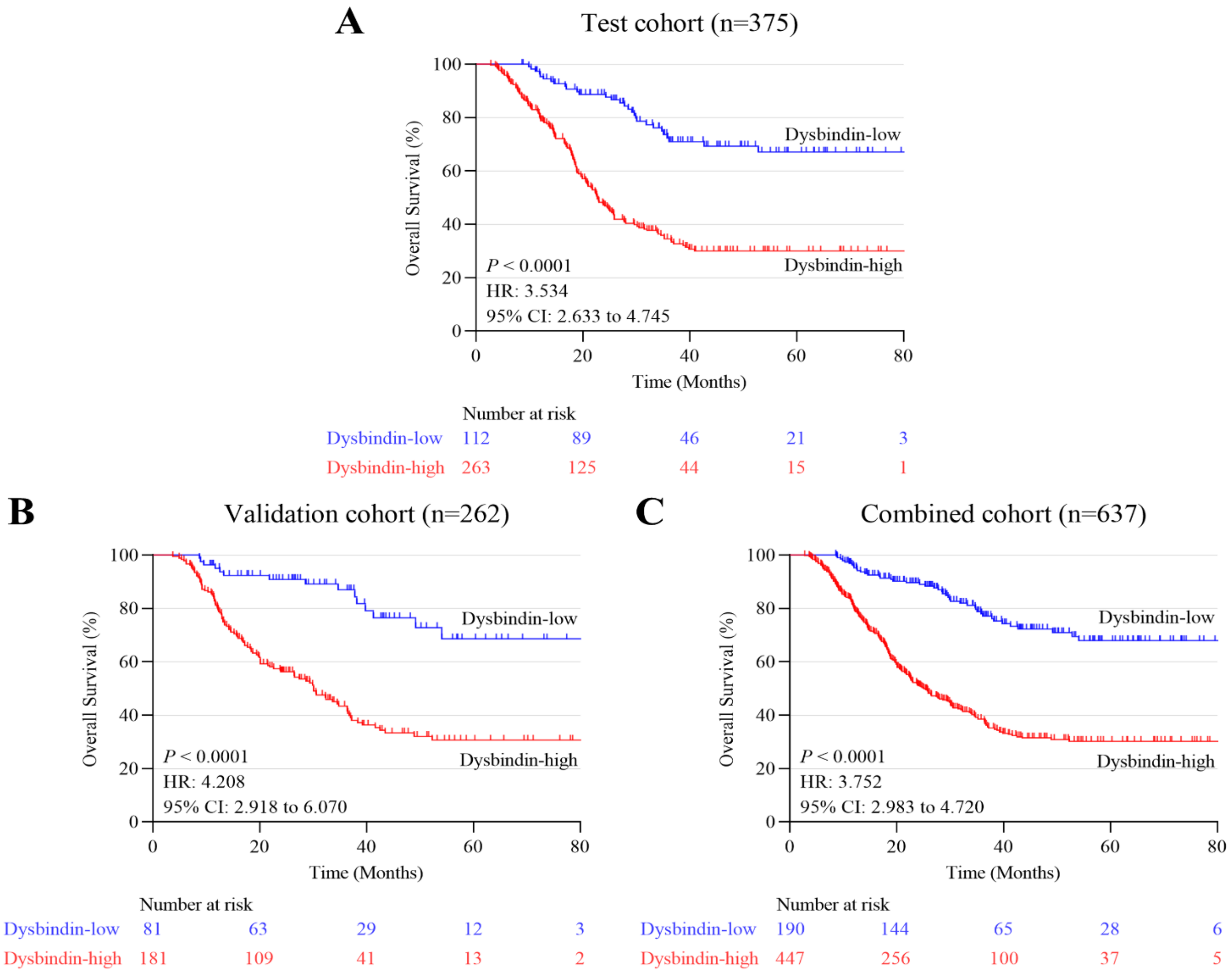

Supplementary Figure 3

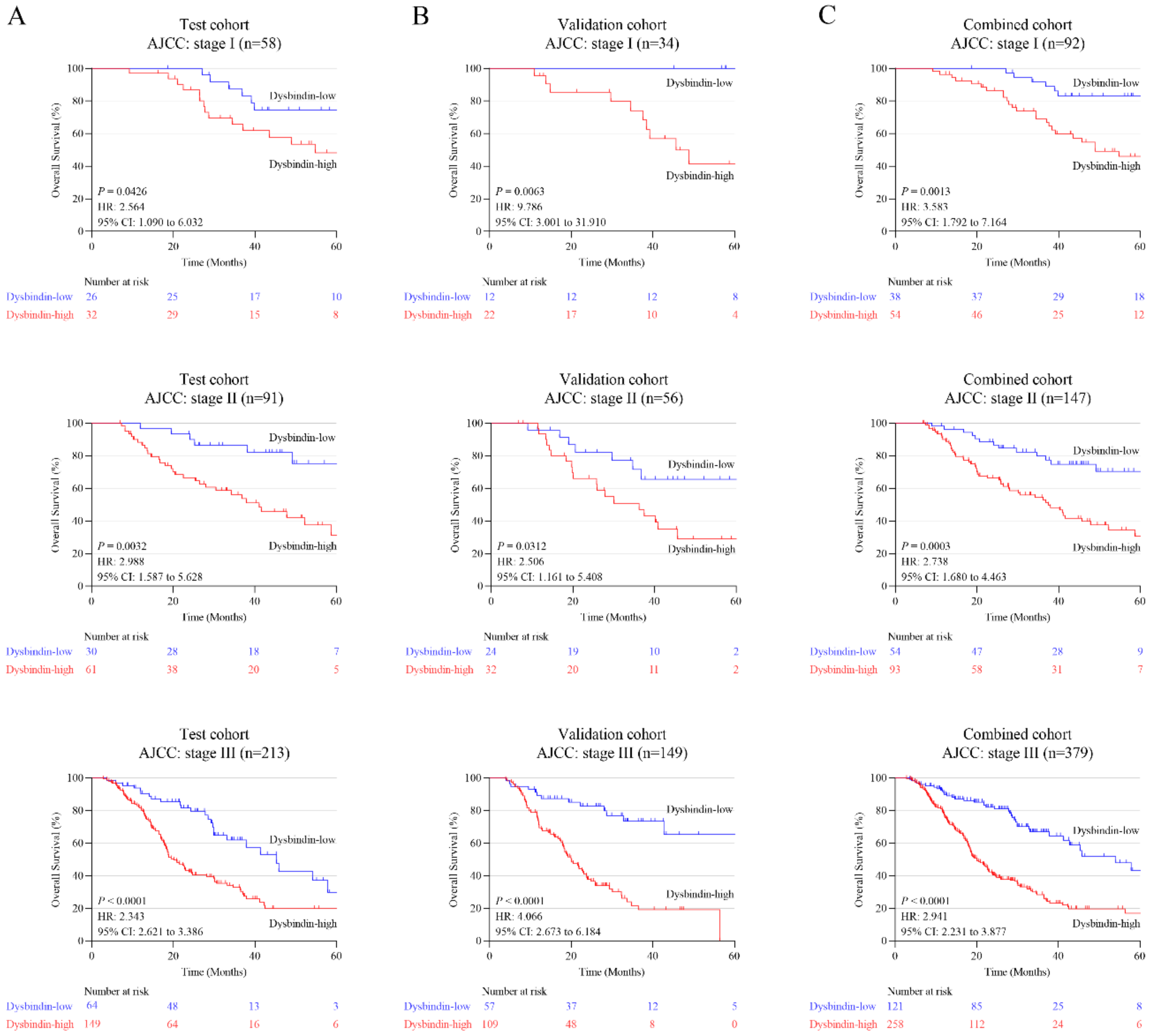

Supplementary Figure 4

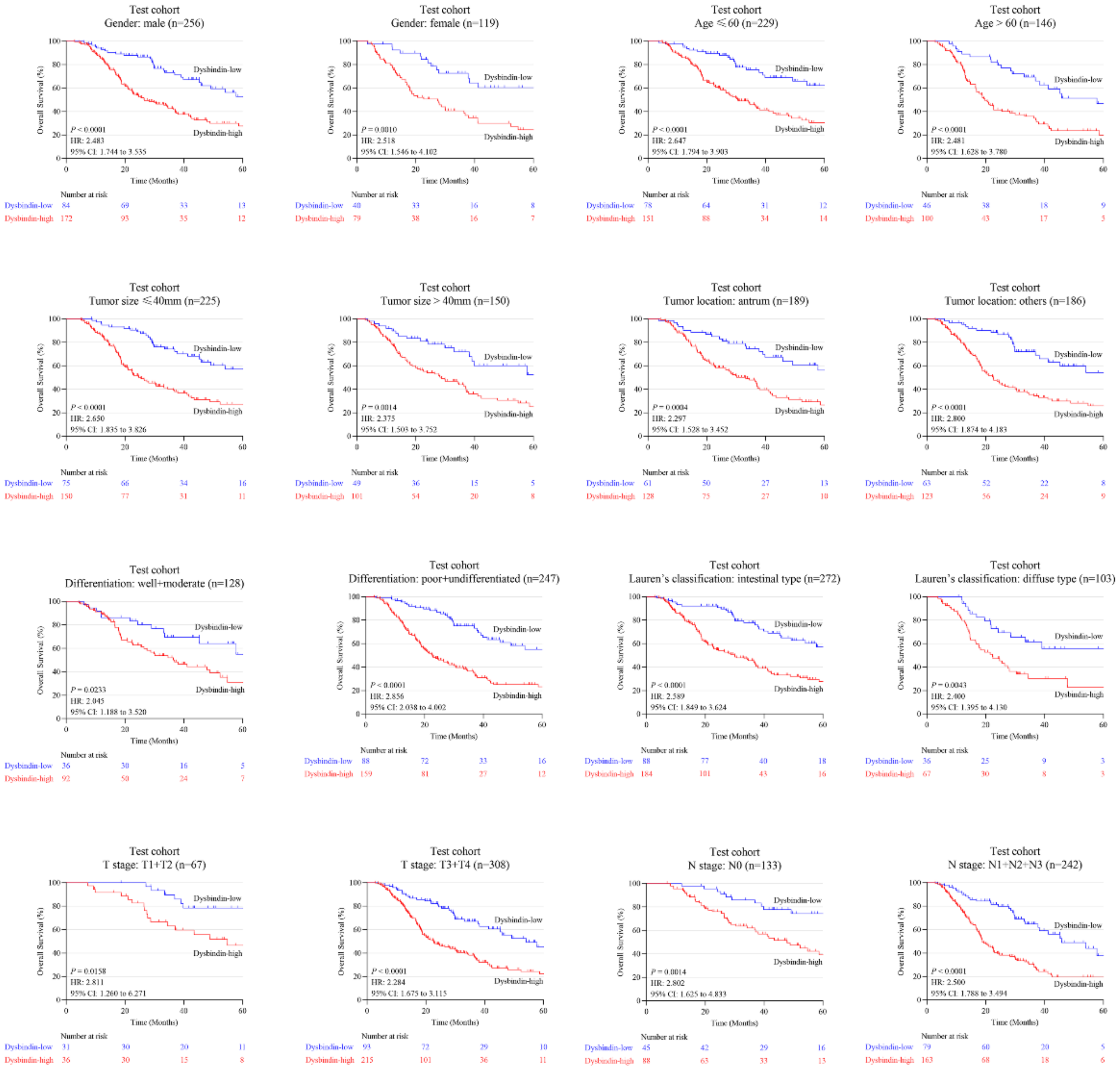

Supplementary Figure 5

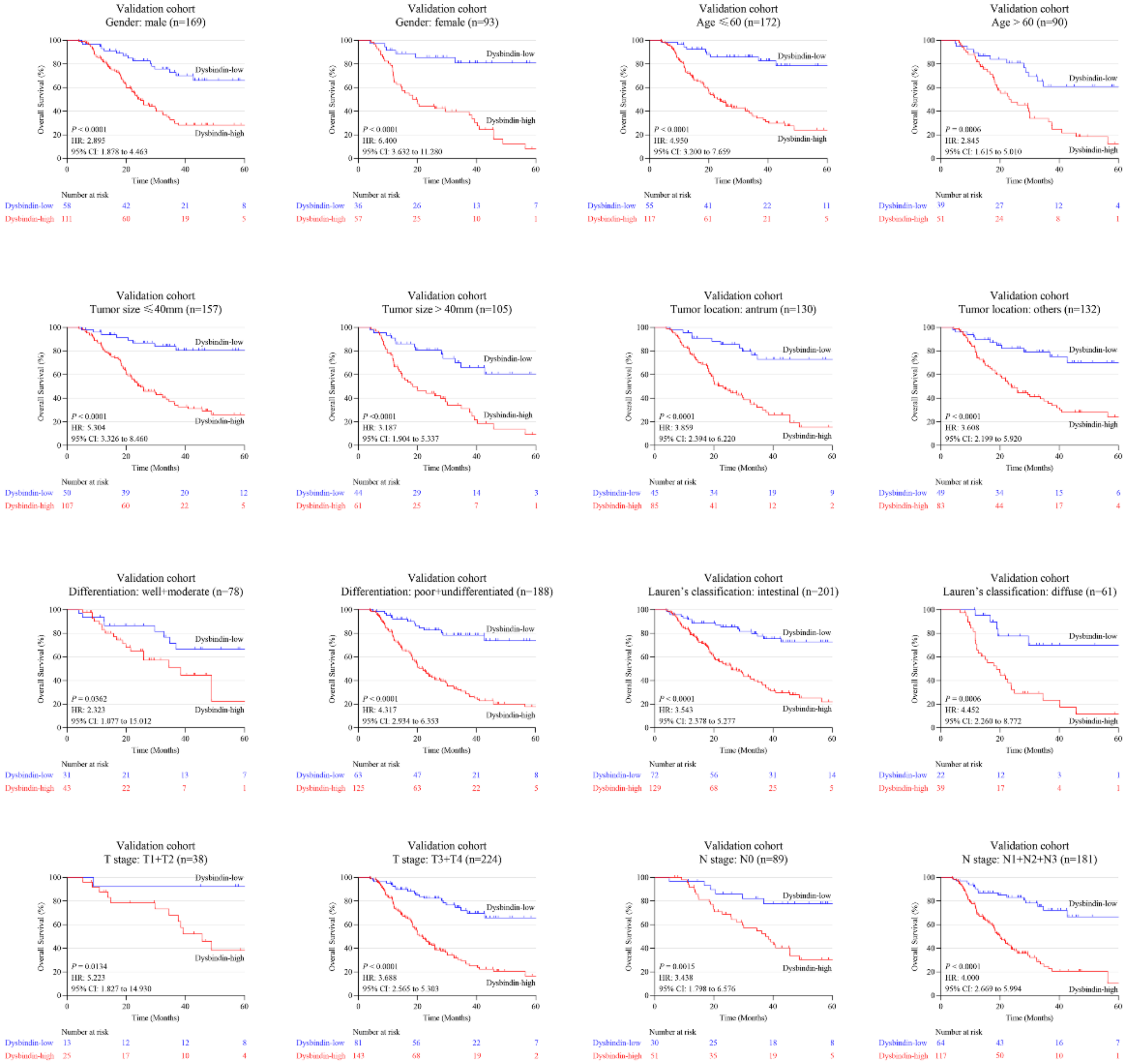

Supplementary Figure 6

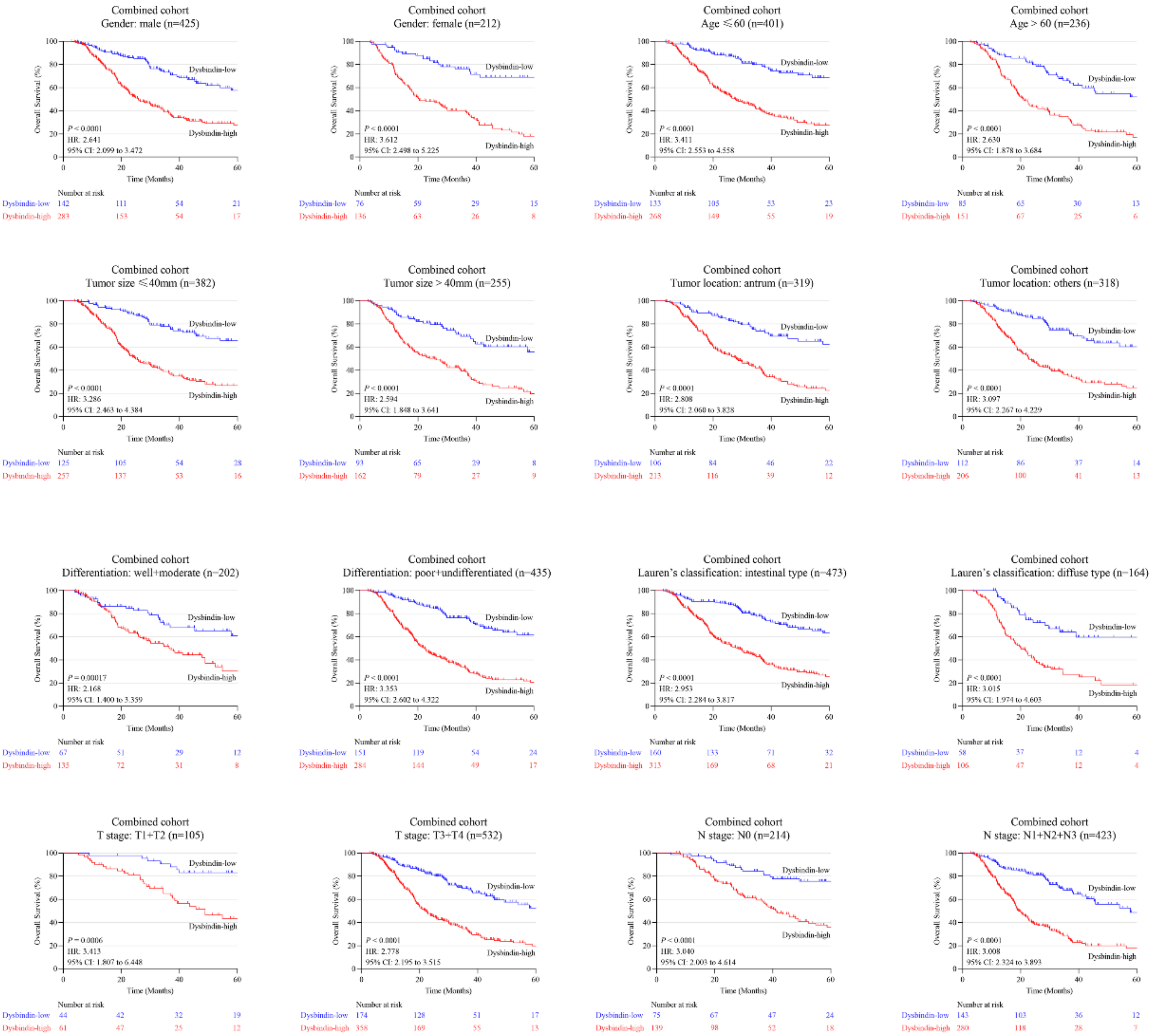

Supplementary Figure 7

A

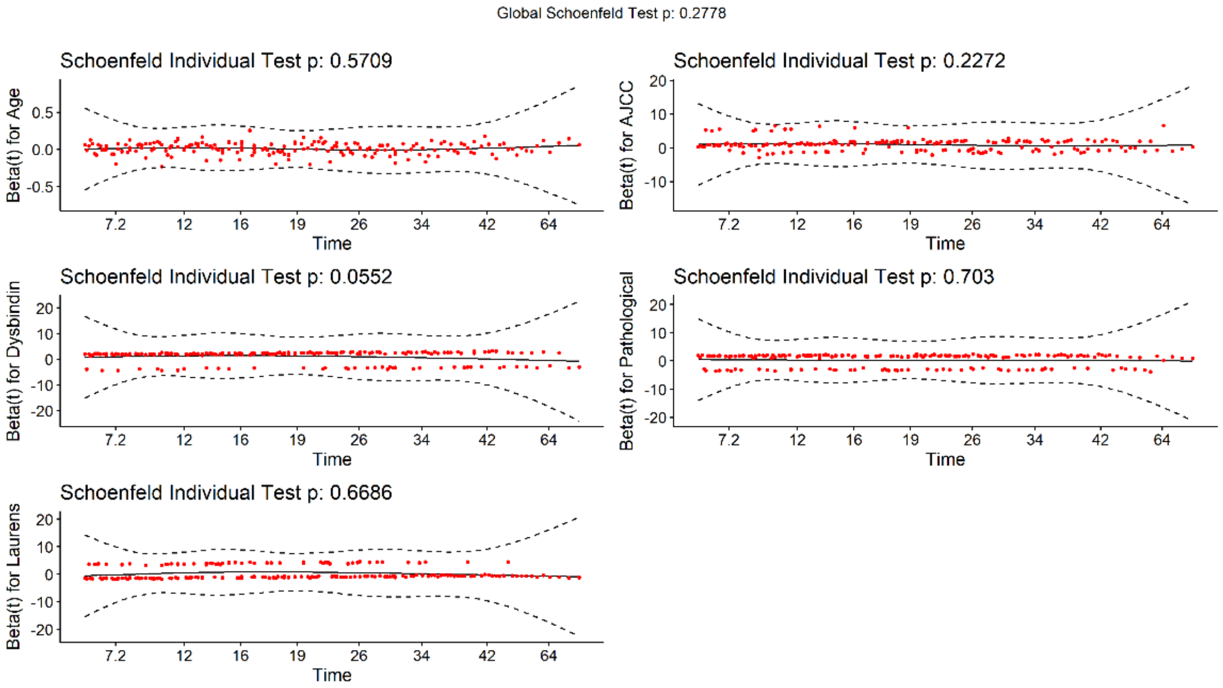

B

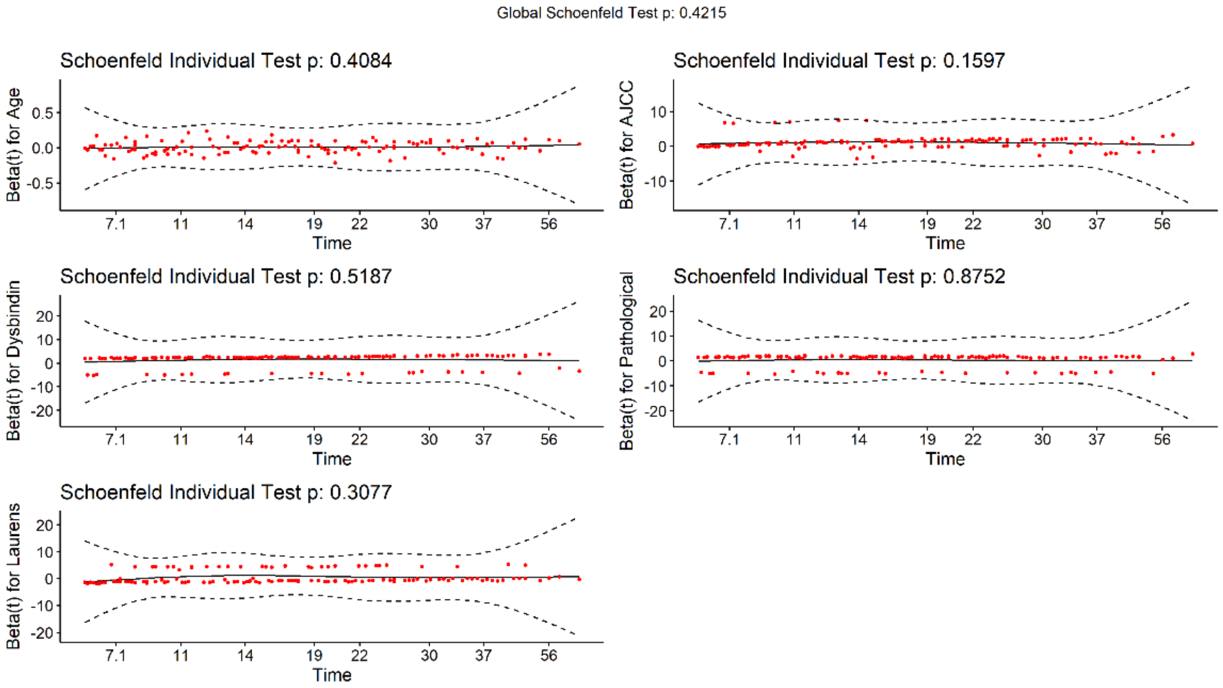

C

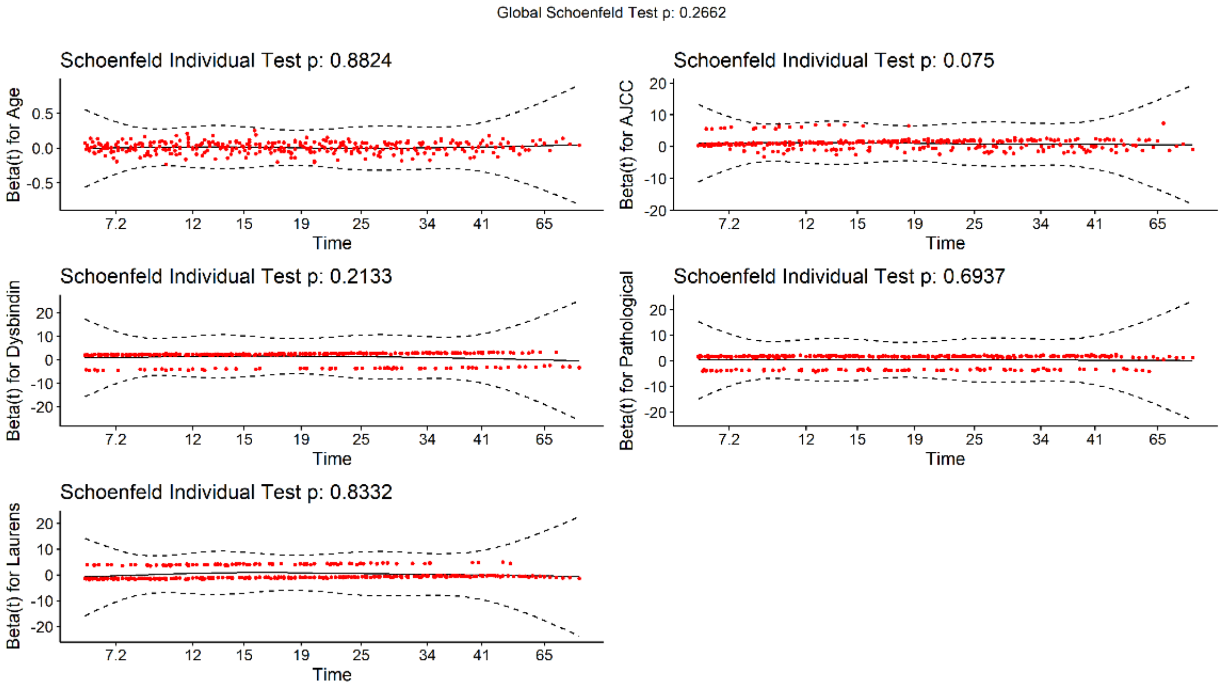

Supplementary Figure 8

A

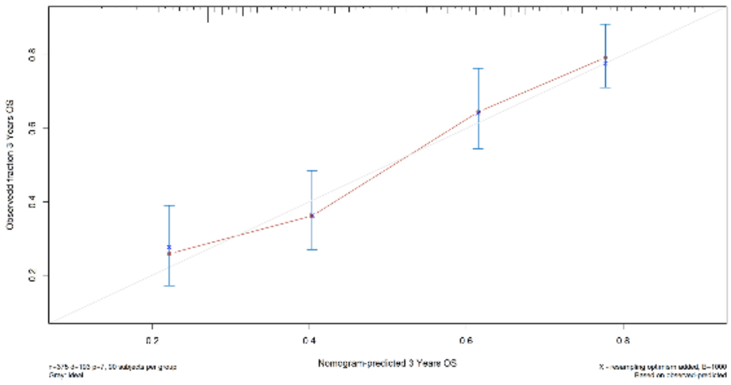

B

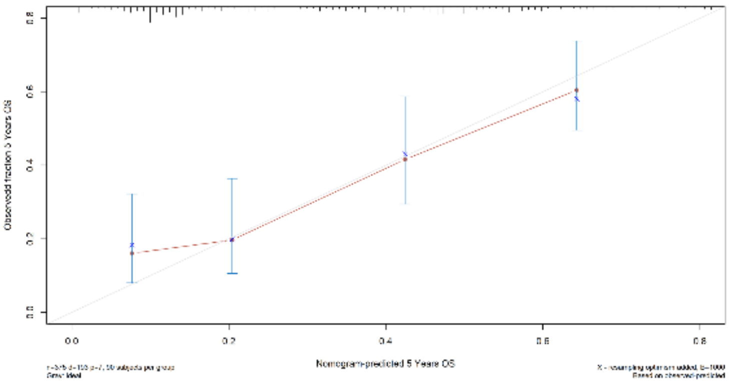

C

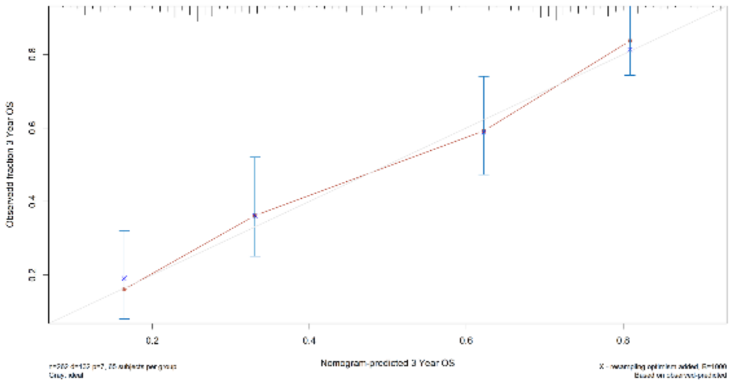

D

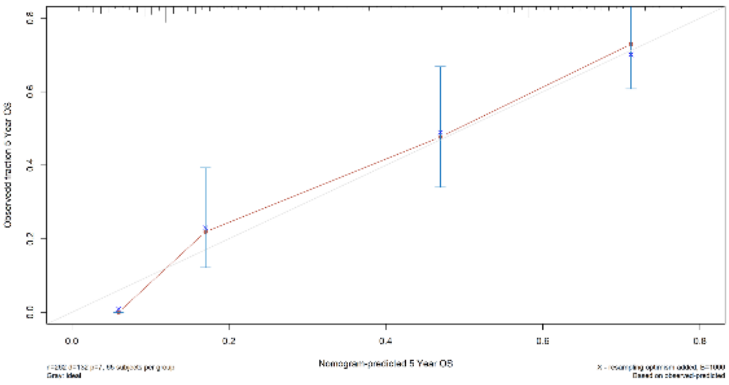

E

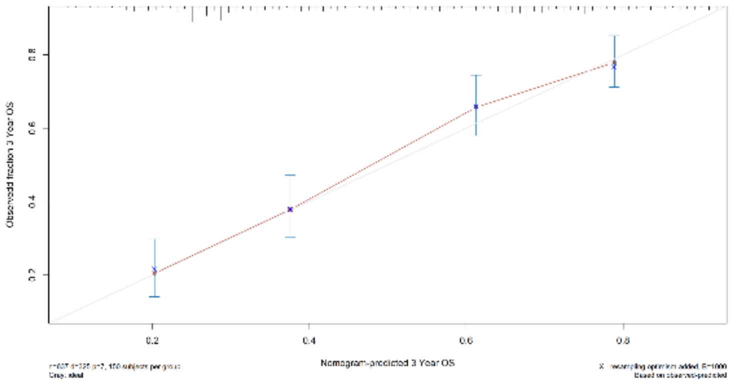

F

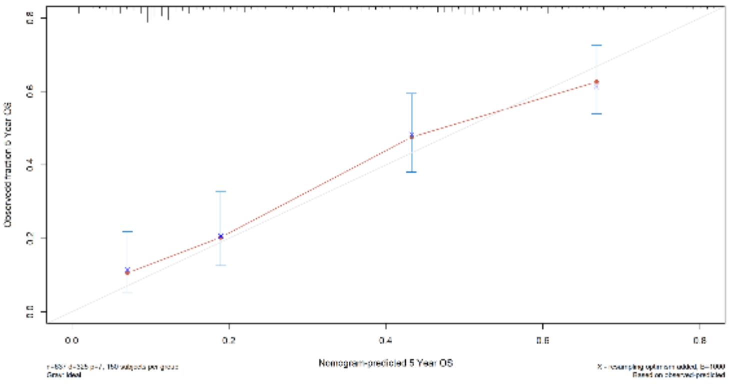

Supplementary Figure 9

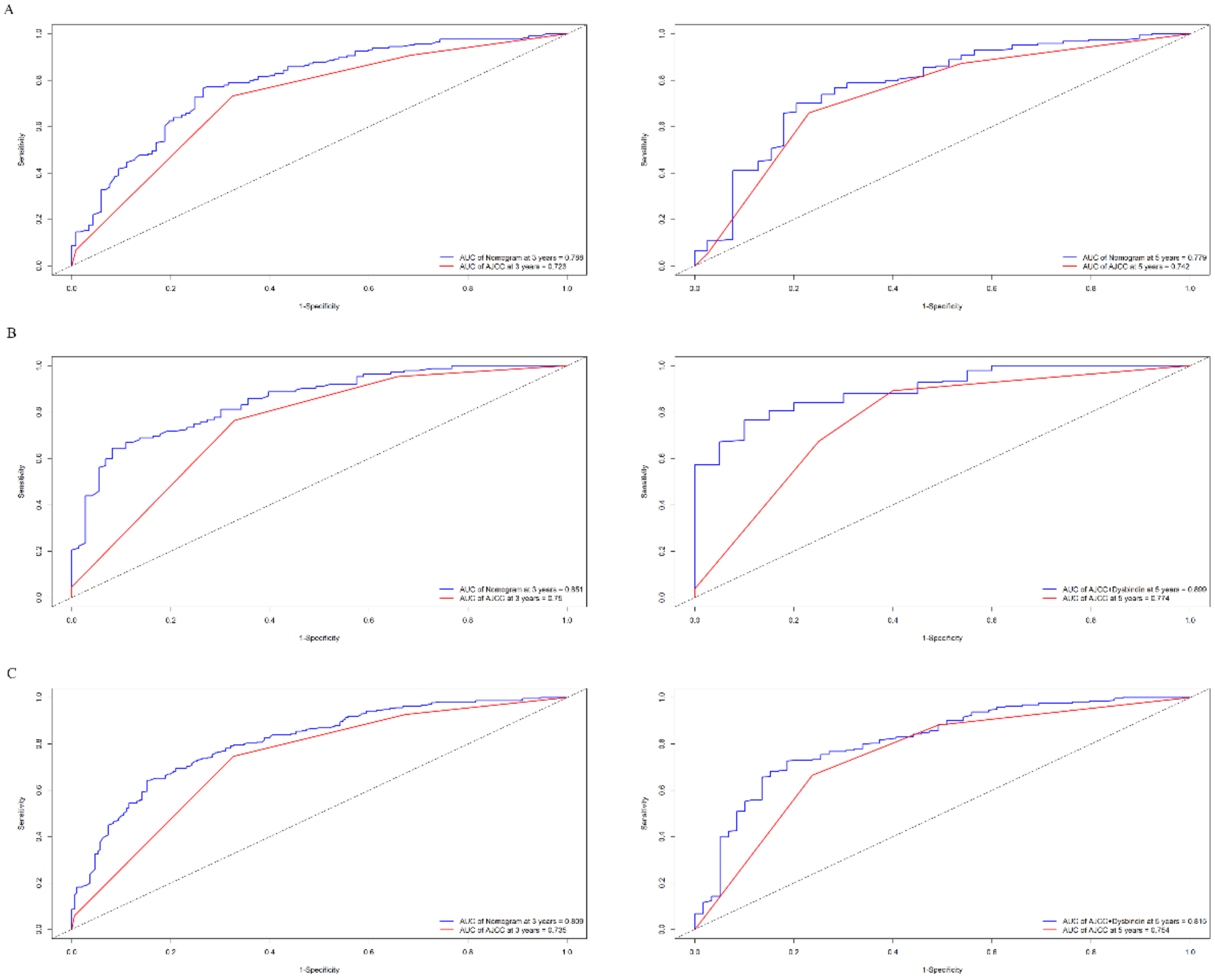

Supplementary Figure 10

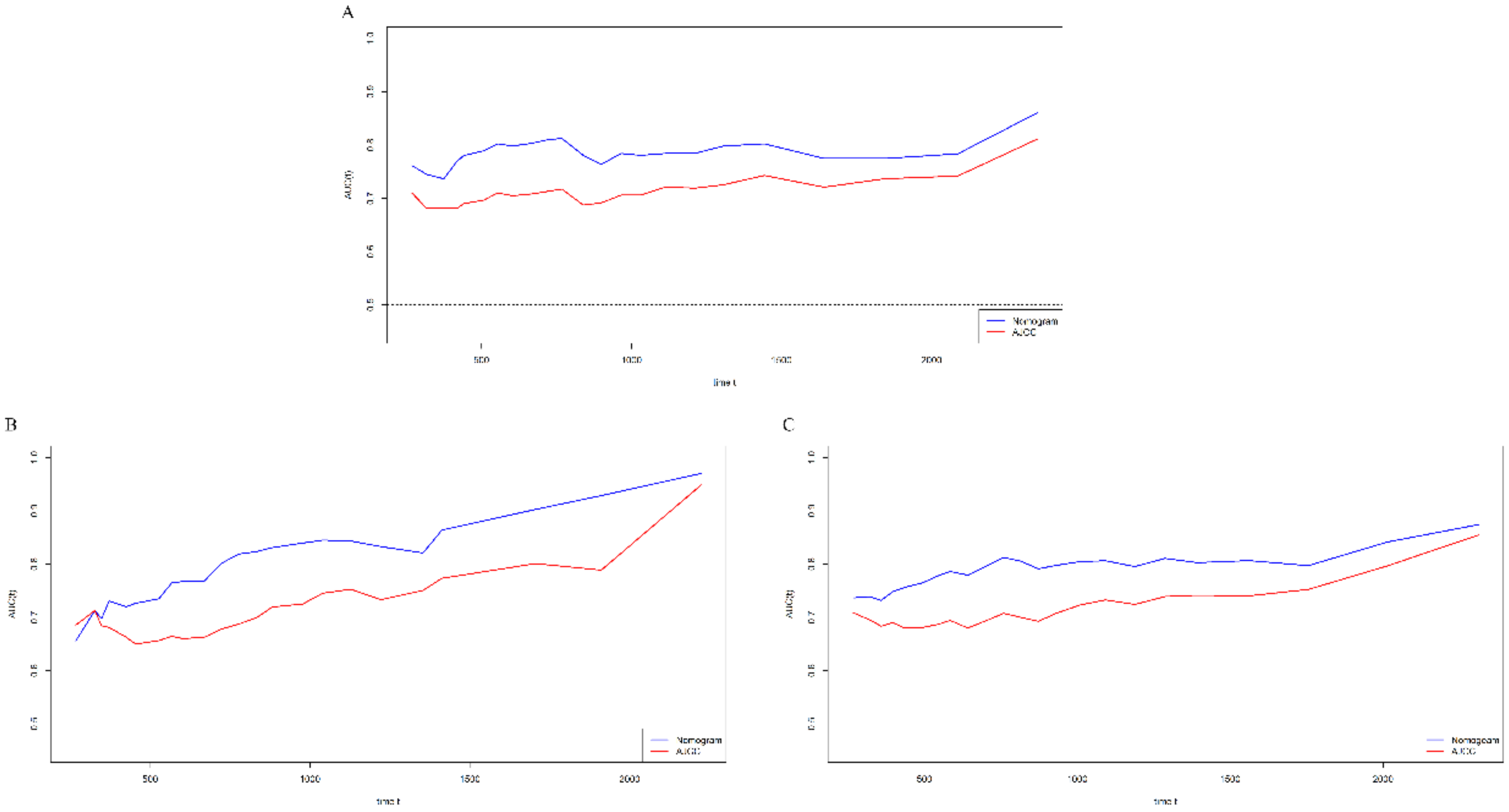

Supplementary Figure 11

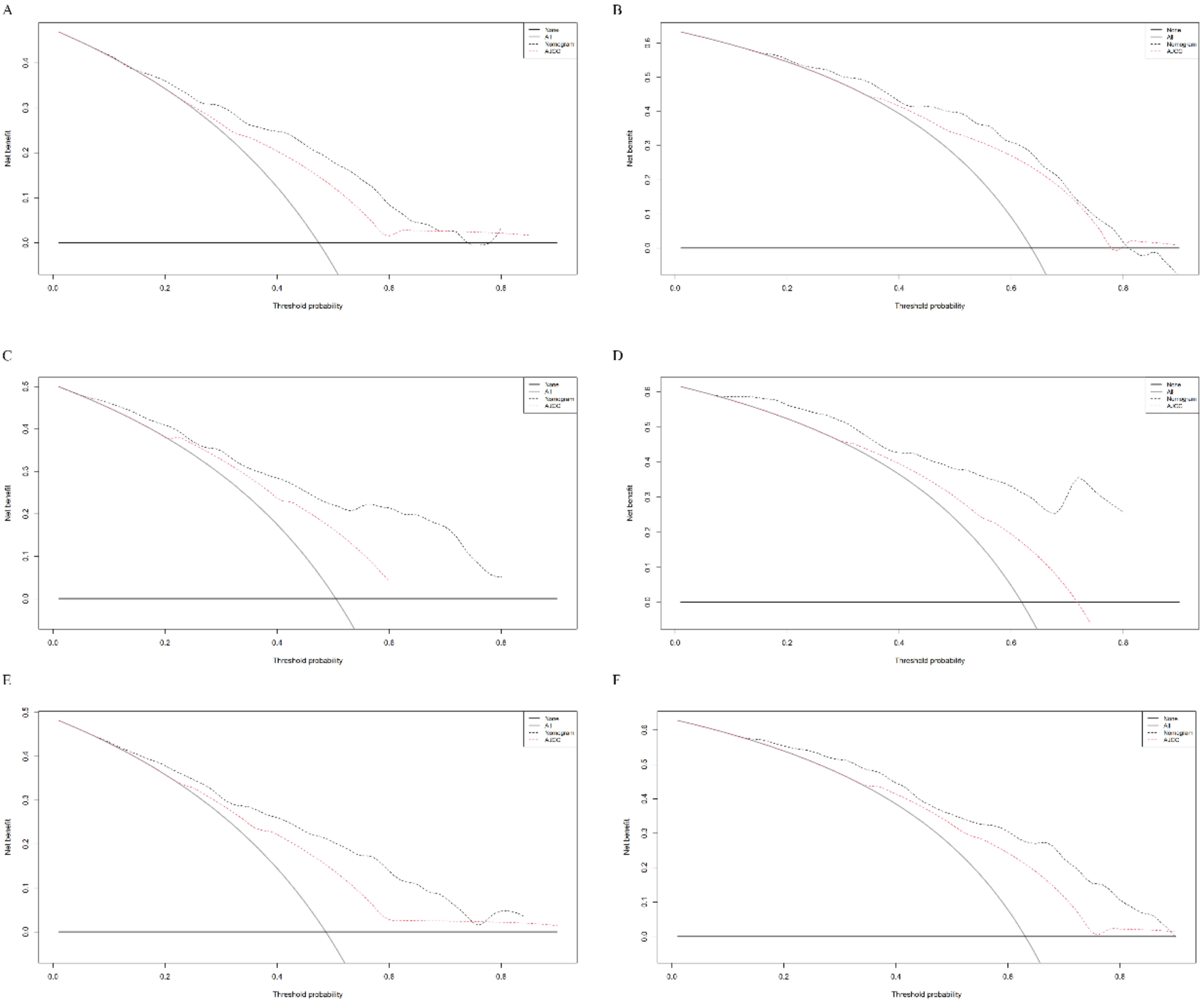

Supplementary Figure 12

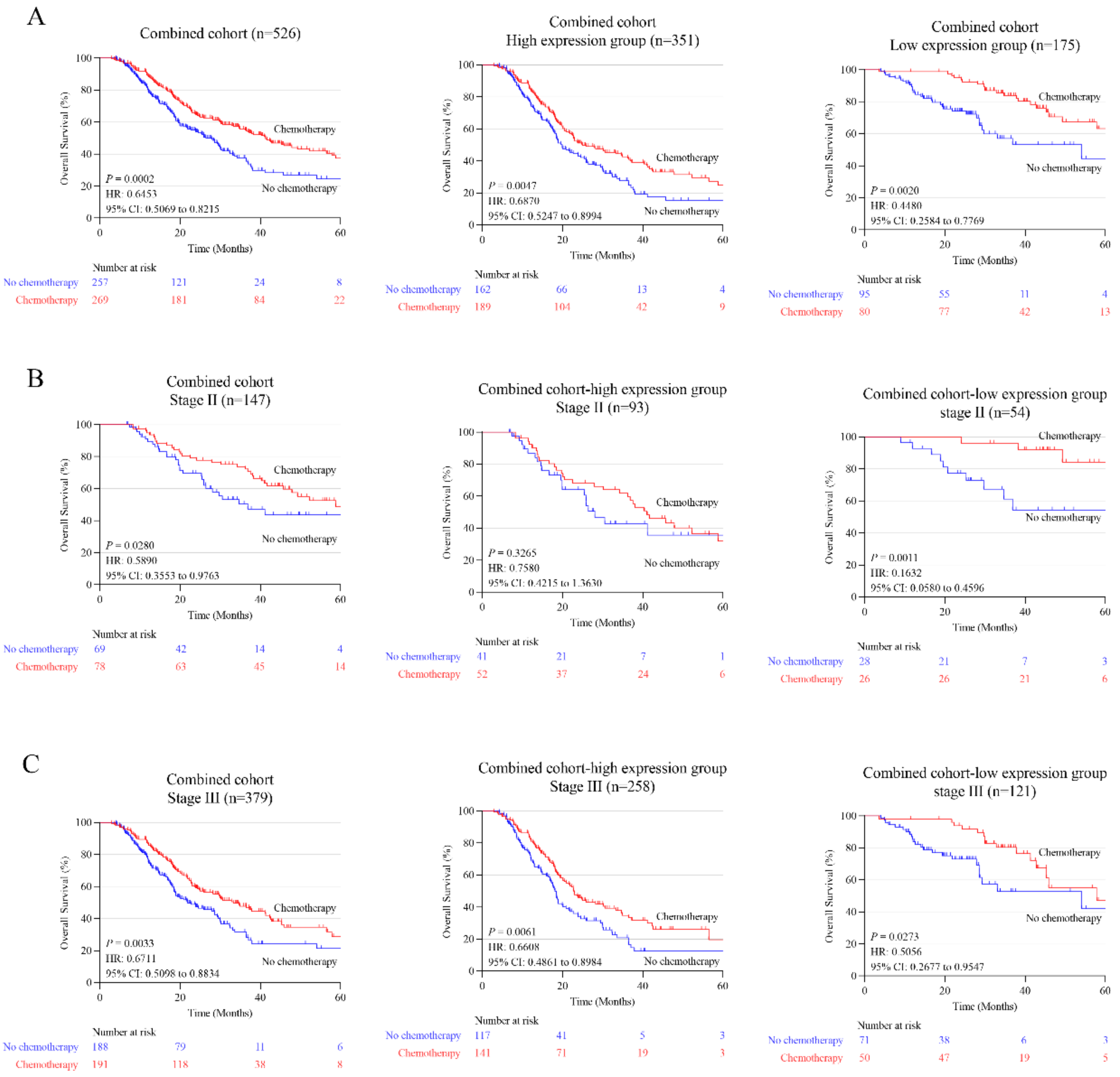

Supplementary Table 1. Clinicopathological characteristics of patients with GAC in test and validation cohorts

Factors	Test cohort		Validation cohort		*P* value
	No	%	No	%	
All patients	375	100	262	100	
Gender					0.321
Male	256	68.3	169	64.5	
Female	119	31.7	93	35.5	
Age (years)					0.239
≤60	229	61.1	172	65.6	
>60	146	38.9	90	34.4	
Size (cm)					0.985
≤4	225	60.0	157	59.9	
>4	150	40.0	105	40.1	
Tumor location					0.504
Cardia	75	20.0	58	22.1	
Body	61	16.3	33	12.6	
Antrum	189	50.4	130	49.6	
Whole	50	13.3	41	15.6	
Pathological differentiation					0.116
Well + moderate	128	34.1	74	28.2	
Poor + undifferentiated	247	65.9	188	71.8	
Lauren’s classification					0.235
Intestinal type	272	72.5	201	76.7	
Diffuse type	103	27.5	61	23.3	
T stage					0.337
T1	41	10.9	18	6.9	
T2	26	6.9	20	7.6	
T3	130	34.7	100	38.2	
T4	178	47.5	124	47.3	
N stage					0.668
N0	133	35.5	81	30.9	
N1	52	13.9	40	15.3	
N2	91	24.3	65	24.8	
N3	99	26.4	76	29.0	
M stage					0.390
M0	362	96.5	256	97.7	
M1	13	3.5	6	2.3	
TNM stage					0.384
I	58	15.5	34	13.0	
II	91	24.3	56	21.4	
III	213	56.8	166	63.4	
IV	13	3.5	6	2.3	
Adjuvant chemotherapy[a]					0.121
Yes	188	50.1	115	43.9	
No	187	49.9	147	56.1	

[a] Patients with GAC received fluorouracil-based chemotherapy for at least 4 cycles.

Supplementary Table 2. Association between dysbindin expression and clinicopathological characteristics of patients with GAC in test and validation cohorts

Factors	Test cohort			Validation cohort		
	**Low**	**High**	**P**	**Low**	**High**	**P**
All patients	124	251		94	168	
Gender			0.878			0.478
Male	84	172		58	111	
Female	40	79		36	57	
Age (years)			0.608			0.069
≤60	78	151		55	117	
>60	46	100		39	51	
Size (cm)			0.893			0.096
≤4	75	150		50	107	
>4	49	101		44	61	
Tumor location			0.926			0.926
Cardia	27	48		22	36	
Body	19	42		11	22	
Antrum	61	128		45	85	
Whole	17	33		16	25	
Pathological differentiation			0.143			0.203
Well + moderate	36	92		31	43	
Poor + undifferentiated	88	159		63	125	
Lauren's classification			0.633			0.972
Intestinal type	88	184		72	129	
Diffuse type	36	67		22	39	
T stage			0.051			0.593
T1	19	22		6	12	
T2	12	14		7	13	
T3	44	86		41	59	
T4	49	129		40	84	
N stage			0.509			0.975
N0	45	88		30	51	
N1	21	31		15	25	
N2	30	61		22	43	
N3	28	71		27	49	
M stage			0.858			0.425
M0	120	242		93	163	
M1	4	9		1	5	
TNM stage			0.209			0.508
I	26	32		12	22	
II	30	61		24	32	
III	64	149		57	109	
IV	4	9		1	5	
Adjuvant chemotherapy[a]			0.073			0.060
Yes	54	134		34	81	
No	70	117		60	87	

[a] Patients with GAC received fluorouracil-based chemotherapy for at least 4 cycles.

Supplementary Table 3. Univariate and multivariate Cox regression analysis of patients with GAC in validation cohort

Factors	Overall survival			
	Univariate analysis		Multivariate analysis	
	**HR (95% CI)**	***P* value**	**HR (95% CI)**	***P* value**
Dysbindin expression		**<0.001**		**<0.001**
Low	1 (Reference)		1 (Reference)	
High	3.858 (2.451-6.072)		3.889 (2.463-6.138)	
Gender		0.650		
Male	1 (Reference)			
Female	1.086 (0.761-1.550)			
Age (years)		0.153		
≤60	1 (Reference)			
>60	1.011 (0.996-1.027)			
Size (cm)		0.073		
≤4	1 (Reference)			
>4	1.370 (0.971-1.934)			
Tumor location		0.738		
Cardia	1 (Reference)			
Body	0.814 (0.427-1.553)			
Antrum	1.094 (0.700-1.709)			
Whole	1.147 (0.657-2.003)			
Pathological differentiation		**0.022**		0.199
Well + moderate	1 (Reference)		1 (Reference)	
Poor + undifferentiated	1.653 (1.076-2.540)		1.328 (0.861-2.048)	
Lauren's classification		0.074		
Intestinal type	1 (Reference)			
Diffuse type	1.432 (0.966-2.122)			
TNM stage		**<0.001**		**<0.001**
I	1 (Reference)		1 (Reference)	
II	2.218 (1.062-4.633)		2.203 (1.051-4.618)	
III	3.822 (1.956-7.465)		3.867 (1.965-7.608)	
IV	25.862 (8.954-74.696)		23.592 (8.122-68.526)	

Supplementary Table 4. Univariate and multivariate Cox regression analysis of patients with GAC in combined cohort

Factors	Overall survival			
	Univariate analysis		Multivariate analysis	
	HR (95% CI)	*P* value	HR (95% CI)	*P* value
Dysbindin expression		**<0.001**		**<0.001**
Low	1 (Reference)		1 (Reference)	
High	2.998 (2.284-3.935)		3.109 (2.365-4.088)	
Gender		0.347		
Male	1 (Reference)			
Female	1.115 (0.888-1.401)			
Age (years)		**0.003**		**0.044**
≤60	1 (Reference)		1 (Reference)	
>60	1.015 (1.005-1.025)		1.010 (1.000-1.019)	
Size (cm)		0.112		
≤4	1 (Reference)			
>4	1.196 (0.959-1.492)			
Tumor location		0.597		
Cardia	1 (Reference)			
Body	0.978 (0.669-1.431)			
Antrum	1.020 (0.763-1.364)			
Whole	1.237 (0.856-1.786)			
Pathological differentiation		**0.007**		**0.013**
Well + moderate	1 (Reference)		1 (Reference)	
Poor + undifferentiated	1.412 (1.100-1.812)		1.376 (1.070-1.769)	
Lauren’s classification		**0.022**		**0.005**
Intestinal type	1 (Reference)		1 (Reference)	
Diffuse type	1.336 (1.043-1.710)		1.436 (1.119-1.844)	
TNM stage		**<0.001**		**<0.001**
I	1 (Reference)		1 (Reference)	
II	1.750 (1.143-2.679)		1.652 (1.078-2.531)	
III	3.336 (2.280-4.881)		3.099 (2.111-4.550)	
IV	14.854 (8.318-26.528)		14.488 (7.985-26.286)	

Supplementary Table 5. Time-dependent ROC curves for dysbindin, TNM staging and clinicopathological characteristics as predictors of deaths within 3-year or 5-year in test, validation and combined cohorts.

Variables	Test cohort	Validation cohort	Combined cohort
	AUC (95% CI)	AUC (95% CI)	AUC (95% CI)
Within 3 year			
Dysbindin + TNM staging	0.765 (0.709-0.821)	0.832 (0.774-0.890)	0.792 (0.751-0.833)
TNM staging	0.723 (0.666-0.780)	0.750 (0.683-0.817)	0.735 (0.692-0.778)
Dysbindin	0.645 (0.590-0.700)	0.691 (0.624-0.758)	0.663 (0.621-0.705)
Age	0.530 (0.461-0.599)	0.537 (0.452-0.622)	0.532 (0.479-0.585)
Gender	0.504 (0.448-0.560)	0.464 (0.394-0.534)	0.488 (0.444-0.532)
Tumor size	0.505 (0.446-0.564)	0.526 (0.462-0.590)	0.513 (0.468-0.558)
Tumor location	0.561 (0.498-0.624)	0.553 (0.474-0.632)	0.530 (0.481-0.579)
Pathological differentiation	0.535 (0.478-0.592)	0.552 (0.488-0.616)	0.544 (0.501-0.587)
Lauren's classification	0.577 (0.528-0.626)	0.570 (0.515-0.625)	0.573 (0.536-0.610)
Within 5 year			
Dysbindin + TNM staging	0.774 (0.687-0.861)	0.889 (0.827-0.951)	0.814 (0.752-0.876)
TNM staging	0.742 (0.657-0.827)	0.774 (0.659-0.889)	0.754 (0.685-0.823)
Dysbindin	0.650 (0.565-0.735)	0.769 (0.663-0.875)	0.692 (0.625-0.759)
Age	0.569 (0.471-0.667)	0.591 (0.454-0.728)	0.574 (0.494-0.654)
Gender	0.483 (0.400-0.566)	0.495 (0.378-0.612)	0.487 (0.419-0.555)
Tumor size	0.542 (0.459-0.625)	0.619 (0.508-0.730)	0.568 (0.503-0.633)
Tumor location	0.561 (0.477-0.645)	0.571 (0.449-0.693)	0.569 (0.500-0.638)
Pathological differentiation	0.496 (0.415-0.577)	0.571 (0.460-0.682)	0.524 (0.459-0.589)
Lauren's classification	0.550 (0.485-0.615)	0.574 (0.498-0.650)	0.558 (0.508-0.608)

Supplementary Table 6. Tests of proportional Hazards assumption in test and validation cohorts

Factors	Test cohort			Validation cohort			Combined cohort		
	**chisq**	**df**	***P* value**	**chisq**	**df**	***P* value**	**chisq**	**df**	***P* value**
Age	0.321	1	0.571	0.684	1	0.410	0.022	1	0.882
AJCC staging	4.338	3	0.227	5.171	3	0.160	6.905	3	0.075
Dysbindin expression	3.677	1	0.055	0.417	1	0.521	1.549	1	0.213
Pathological differentiation	0.145	1	0.703	0.025	1	0.883	0.155	1	0.694
Lauren’s classification	0.183	1	0.669	1.041	1	0.311	0.044	1	0.833
Global test	8.663	7	0.278	7.071	7	0.422	8.815	7	0.266

Supplementary Table 7. Comparison of the C-index of nomogram and TNM staging between test, validation and combined cohorts

Variables	C-index	95% CI	P value
Test cohort			<0.001
nomogram	0.720	0.7004-0.7396	
TNM staging	0.646	0.6127-0.6793	
Validation cohort			<0.001
nomogram	0.718	0.6729-0.7631	
TNM staging	0.632	0.5928-0.6712	
Combined cohort			<0.001
nomogram	0.719	0.6916-0.7464	
TNM staging	0.641	0.6155-0.6665	

[a]included stage II and III patients with GAC
